# Characterizing Oxide Inclusions in Welded Lean Duplex Stainless Steels and Their Influence on Impact Toughness

**DOI:** 10.3390/ma16051921

**Published:** 2023-02-25

**Authors:** Suphitcha Moonngam, Pranpreeya Wangjina, Ekkarut Viyanit, Chaiyasit Banjongprasert

**Affiliations:** 1 Department of Physics and Materials Science, Faculty of Science, Chiang Mai University, 239 Huay Kaew Road, Chiangmai 50200, Thailand; 2 Graduate Program in Materials Science, Faculty of Science, Chiang Mai University, 239 Huay Kaew Road, Chiangmai 50200, Thailand; 3 National Science and Technology Development Agency, 114 Thailand Science Park, Paholyothin Road, Klong Luang, Pathum Thani 12120, Thailand; 4 Center of Excellence in Materials Science and Technology, Chiang Mai University, 239 Huay Kaew Road, Chiangmai 50200, Thailand

**Keywords:** lean duplex stainless steel, oxide inclusions, flux-cored arc welding, impact testing

## Abstract

In newly developed 2101 lean duplex stainless steel, oxide inclusions have been detected on welded metal zones after subjecting them to flux-cored arc welding with an E2209T1-1 flux-cored filler metal. These oxide inclusions directly affect mechanical properties of the welded metal. Hence, a correlation requiring validation has been proposed between oxide inclusions and mechanical impact toughness. Accordingly, this study employed scanning electron and high-resolution transmission electron microscopy to assess the correlation between oxide inclusions and mechanical impact toughness. Investigations revealed that the spherical oxide inclusions comprised a mixture of oxides in the ferrite matrix phase and were close to intragranular austenite. The oxide inclusions observed were titanium- and silicon-rich oxides with amorphous structures, MnO with a cubic structure, and TiO_2_ with an orthorhombic/tetragonal structure, derived from the deoxidation of the filler metal/consumable electrodes. We also observed that the type of oxide inclusions had no strong effect on absorbed energy and no crack initiation occurred near them.

## 1. Introduction

During arc welding, such as flux-cored arc [1], gas metal arc [2,3], and submerged arc welding [4,5], oxide inclusions are formed due to the oxidation and solidification of molten metals. These oxides are derived from oxygen via the reaction with elements from fluxes, filler metals in the welded metal zone [5,6,7], vaporized metal, and shielding gases (such as carbon dioxide) [3]. Interestingly, these inclusions strongly influence the corrosion behavior of stainless steel, such as pit initiation by oxide inclusions, favoring the formation of a sufficient number of inclusions [6,8]. Furthermore, they degrade the mechanical properties of stainless steel by creating voids around inclusion-generating microcracks [6]. Moreover, in welding, they directly affect mechanical properties, as their sufficient size and quantity make weldment more brittle [9]. Hence, these oxide inclusions have been attributed to two types: particles and films. Studies have also reported that while oxide inclusions have little effect on weld quality when the oxide inclusions are small and take the form of oxide particles [10], large oxide inclusions (>1 µm) result in poor toughness due to stress concentration and induced cracks [11], crack initiation and growth [6,12], and pitting corrosion [6]. For example, titanium-rich oxide inclusions were commonly found to cause ferrite nucleation [13] and stimulate the nucleation of acicular ferrite in welds [2,14], increasing the toughness [15,16] and improving the ductility of welds [16].

A study reported that oxide inclusions, such as titanium- and silicon/manganese-rich oxides, commonly exist in a nearly spherical shape [17], with TiO_2_ inclusions being prominent during flux-cored arc welding processes due to the high proportion of TiO_2_ particles in the slag system [17]. The most common oxide inclusions in stainless steels are Al_2_O_3_, TiO_x_, Cr_2_O_3_, SiO_2_, MnO, and Ti_3_O_5_ [6,9], including manganese- or silicon- and titanium-rich oxides (such as MnTi_2_O_4_)—the latter of which are associated with gas metal [2,18] and flux-cored arc welding [18,19]. However, although oxide inclusions strongly influence weld properties regarding the size, quantity, shape, and type of oxide inclusions, as mentioned above, and lean duplex stainless steels have recently been attractive in the stainless steel market due to their high mechanical properties and corrosion resistance [20], only few studies on the oxide inclusions of welded lean duplex stainless steels and their relationships to mechanical properties exist. In addition to that, a comprehensive study on oxide inclusions in welded 2101 lean duplex stainless steel is yet to be conducted, including investigations on the characteristics of oxide inclusions in the newly developed 2101 lean duplex stainless steel welded by E2209T1-1. Therefore, this paper characterized and identified the types of oxide inclusions generated from the E2209T1-1 flux-cored filler metal of newly developed 2101 lean duplex stainless steel welds. Then, we used a high-resolution transmission electron microscope to characterize the oxide inclusions in these welded samples, followed by an investigation of oxide inclusion effects on metal-based mechanical properties via impact testing. Their influence on the impact toughness of the welded joints can be observed because the adverse effect of oxide inclusions directly impacts the toughness, mainly on the welded metal zone due to the filler metal.

## 2. Results and Discussion

**Figure 1:**
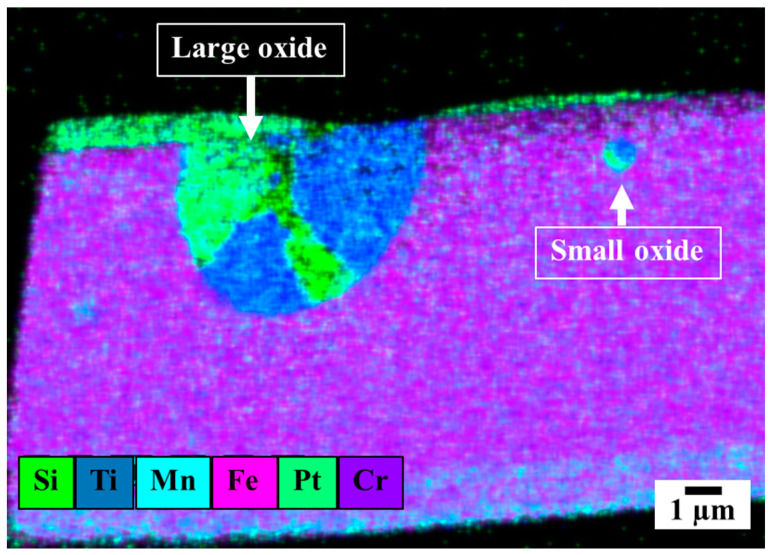
Elemental distributions of the oxide inclusion in the welded metal 2101 joint by EDS-TEM.

Figure 1 shows that the oxide inclusions were typically almost spherical, possessing inhomogeneous alloying elements similar to those previously reported [17]. Specifically, the specimens comprised two different oxide inclusions (large and small oxides rich in silicon, titanium, and manganese), as shown in Figure 1, and the spherical oxide inclusions had little detrimental effects on the weld quality because they were small particles and not oxide films [10]. Our investigations also revealed that the carbon dioxide shielding gas was preferred for welding mild- and low-alloy steels, acting as an oxidizing agent for wide and deep joint penetration. However, the welding time was short (~2 min per pass), leading to a short time for vaporized metals to react with oxygen in the weld pool.

**Figure 2:**
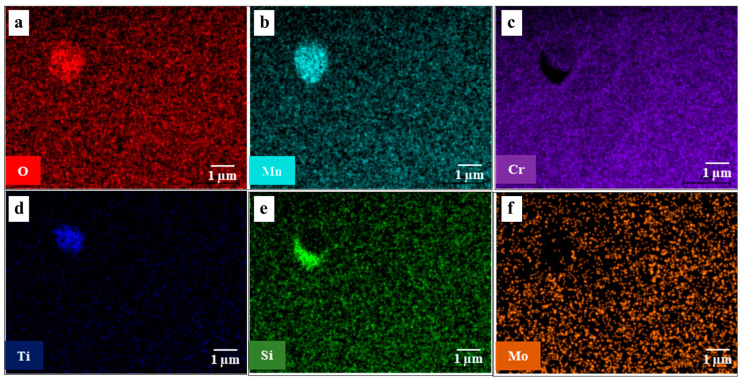
Elemental distribution of small oxide inclusions by EDS-TEM: (**a**) oxygen; (**b**) manganese; (**c**) chromium; (**d**) titanium; (**e**) silicon; (**f**) molybdenum.

According to Figure 2 and Table 1, the oxide particles were enriched with different elements, such as manganese, titanium, silicon, and chromium. Notably, although the oxygen distribution in the oxide inclusions was mainly associated with manganese and silicon, such inclusions were derived from the flux [21] in the E2209T1-1 filler metal. The composition of the E2209T1-1’s flux had an important effect on the oxide inclusion type due to the high content of titanium and silicon.

**Figure 3:**
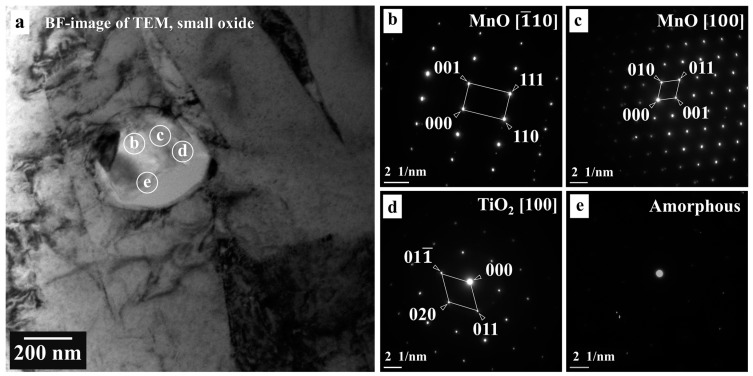
TEM images corresponding to small oxide inclusions: (**a**) BF-image with selected areas for diffraction patterns; (**b**,**c**) SADP of MnO; (**d**) SADP of TiO_2_; (**e**) and SADP of amorphous.

**Figure 4:**
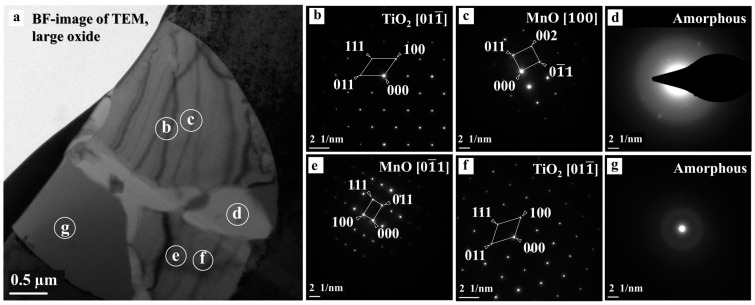
TEM images corresponding to large oxide inclusions: (**a**) BF-image with selected areas for diffraction patterns; (**b**) SADP of TiO_2_; (**c**) SADP of MnO; (**d**) SADP of amorphous; (**e**) SADP of MnO; (**f**) SADP of TiO_2_; (**g**) and SADP of amorphous.

Conversely, Figure 3 and Figure 4 show that since the oxide inclusions located on the ferrite matrix (large oxide) were close to intragranular austenite (small oxide), the oxide inclusion was formed due to intragranular austenite [19]. We relate this identification to the fact that oxide inclusions play a crucial role in acicular ferrite nucleation [17]. Figure 3 and Figure 4, however, show the BF images of TEM, the oxide phases, and the areas analyzed by EDS point analysis and SADP. We observed that the oxide’s SADP matched that of the TiO_2_ [$01\bar{1}$] orthorhombic structure, TiO_2_ [100] orthorhombic structure, and the TiO_2_ [$01\bar{1}$] tetragonal structure, including MnO [100], MnO [$\bar{1}10$], and MnO [$0\bar{1}1$], indicating that the formation of a titanium-rich oxide, acting as a nucleation site, formed the silicon- or manganese-rich oxides [17]. Alternatively, we identified amorphous manganese- and silicon-rich oxides in Figure 3e and Figure 4d,g. Since MnO has a cubic structure, and TiO_2_ has multiple crystalline forms, including tetragonal (rutile and anatase) and orthorhombic structures (brookite), titanium and manganese oxides are commonly found in welded metals [5]. Accordingly, a study reported that titanium-rich oxides were the first generated on an interface by gas metal arc welding [14]. Similarly, we observed that while the inclusions mainly comprised an oxide layer with cubic MnO, including orthorhombic and tetragonal TiO_2_ with different zone axes, some areas contained manganese- or silicon-rich oxides with an amorphous structure resulting from the filler metal, which agrees with previous studies [1,5,7]. We also observed that both amorphous manganese oxide and titanium oxide inclusions occurred, agreeing with previous findings on the flux types of titanium oxide with some silicate [22]. However, this finding contrasts with [2], where the complex oxide inclusions were characterized as spherical MnTi_2_O_4_ by SADP, causing this phase to promote acicular ferrite nucleation [2].

Table 2 shows that although 2101 lean duplex stainless steel base material has a high impact toughness at 121.0 ± 1.4 J at room temperature, this study’s absorbed impact energy of the flux-cored arc welded joints was 32.7 ± 0.6 J. This finding indicates that while the absorbed energy from the impact testing was comparatively low compared to previous reports, the absorbed energy was higher than that of the subsize welded duplex stainless steel specimen at 16 J, according to ASTM A370-21. Investigations also revealed that this study’s absorbed welded joint energy after flux-cored arc welding was comparable with the other welded metal with a 2101/2101 welded joint by E2209 (39.6 ± 0.7 J) [12], as summarized in Table 2. Accordingly, a study reported that some large oxide inclusions existed in the welded metal that decreased the impact toughness of the investigated welded metal, even though the normal size distribution of oxide inclusions was small [7]. In contrast, we observed that although the percentage of large oxide inclusions (>1 µm) was 14.2%, the percentage of small oxide inclusions (<1 µm) was 85.8%, but the maximum size of oxide inclusions in the welded metal was 7.3 µm. This finding indicates that the impact toughness of the welded metal decreases where large oxide inclusions are produced. Previously, Pu et al. [4] reported that the toughness of the weld metal would be reduced if there was an increase in the amount and size of inclusions [4], with another study reporting a relationship between the oxide inclusions in the welded metal zone and the high content of CO_2_ shielding gas [3], and an impairment of the impact toughness of welds being caused by the oxide inclusions as reported by [23].

**Figure 5:**
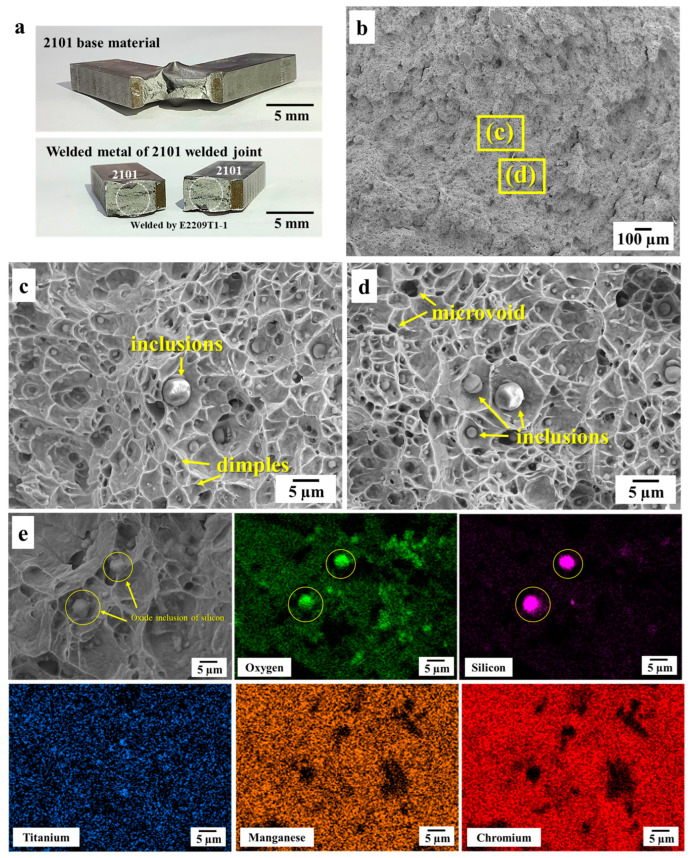
Schematic showing the (**a**) fracture location after the impact test; (**b**) fracture surface micrographs of the welded joints at 60× magnification with areas of interest (**c**,**d**); (**c**,**d**) fracture surfaces at 2000× magnification; (**e**) SEM fracture surfaces with EDS mapping.

Alternatively, Figure 5 shows the fracture surface with a ductile fracture mode and dimples due to their small size and uniform oxide inclusion distributions [7]. Although the TiO_2_ particles identified by SADP/TEM in which TiO_2_ was not dominated but improved the toughness of the weld metal zone was observed, this finding contrasts with a previous finding [15]. Furthermore, investigations revealed that while the main feature of the fracture surface was fine dimples, no apparent crack initiation and propagation sites were observed in these welded joints near oxide inclusions. In contrast to [11], however, large oxide inclusions (1–18 µm) that were spherical were formed inside the weldment, leading to the linking of initial fracture with microcracks [6,11]. Studies have reported that the impact toughness of a weld decreases due to the number of cleavages and small dimples surrounding the inclusions [12,24], which was not the case for this research. In addition, large oxide inclusions (1–5 µm) from FCAW that resulted in a discontinuous fracture, lowering the welded metal’s impact toughness by 2205/2205 and that of welded joints by E2209, were reported by another study [25]. It has also been reported that the absorbed energy of the welded metal by E2209 (32.7 ± 0.6 J) was higher than that of the welded metal by E2101 at 27 J (for the subsize specimen) [26]. Therefore, since the impact toughness of the welded joint was lower than that of base materials in terms of welding, we propose its suitability for welding applications within an acceptable range of ASTM A370-21.

**Table 2:** Results after impact testing using the impact-absorbed energy from the tested specimens.

Materials	Absorbed Energy (J)
Welded 2101 metal joint by E2209 under FCAW	32.7 ± 0.6
2101 base material	121.0 ± 1.4
2101 base material tested at 20 °C [26]	133
2101 base material tested at −40 °C [12]	96.0 ± 8.0
Welded 2101 metal joint by E2209 under hyperbaric FCAW tested at −40 °C [12]	39.6 ± 0.7

## 3. Materials and Methods

**Figure 6:**
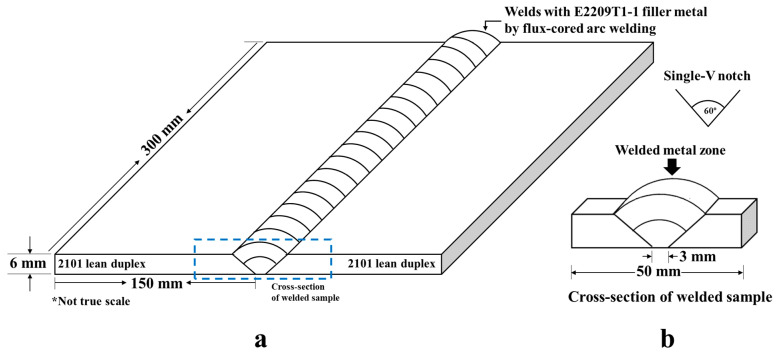
Schematic showing (**a**) the welded 2101 lean duplex stainless steel sample using flux-cored arc welding and (**b**) a cross-section of the weld samples for FIB/TEM at the welded metal zone.

As shown in Figure 6, the welded samples were made of 2101 lean duplex stainless steel (Outokumpu UNS S32101) (with a thickness of 6 mm) via flux-cored arc welding with an E2209T1-1 filler (Selectarc, AWS A5.22) [7]. Our welding parameters were 200 A, 25 V, and a welding speed of 35 cm/min for the weld root and 30 cm/min for the weld cap, with CO_2_ as the shielding gas, as reported previously [7]. Table 3 lists the chemical compositions of the stainless steel and filler metal.

**Figure 7:**
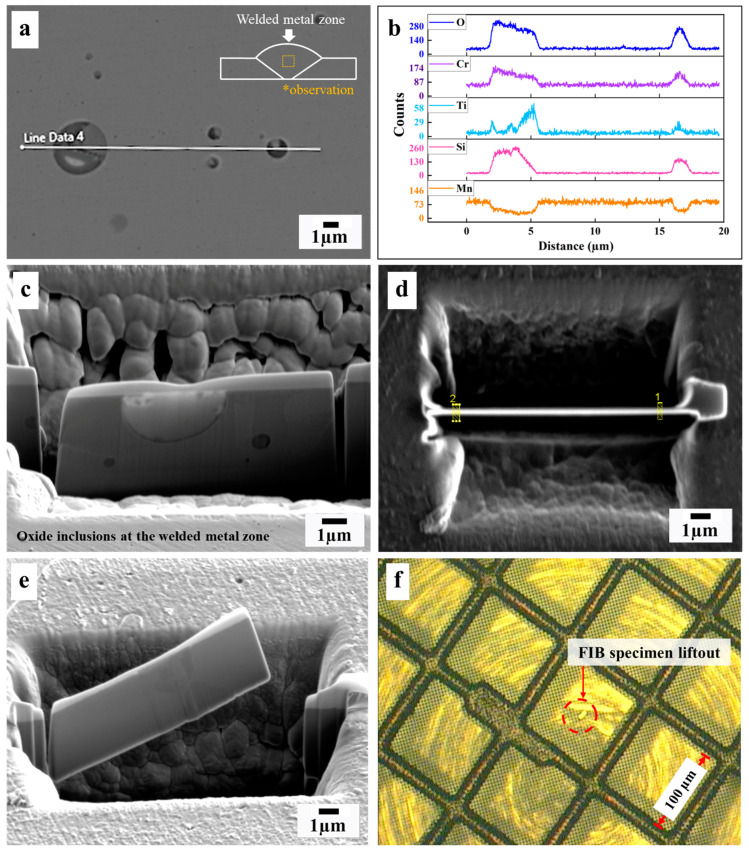
Schematic showing the cross-sectional positions of the welded metal zone after (**a**) SEM; (**b**) line scanning on the oxide inclusions; (**c**–**e**) oxide inclusions cut off by FIB; (**d**) sequence of cutting (1, 2); (**f**) specimen lift-out and adhesive on the copper grid.

First, we ground and polished the cross-sectioned weld samples, following metallurgical sample preparation. Then, a focused ion beam (FIB, FEI Nova Nanolab 200) with a combined dual beam and a scanning electron microscope (SEM) was used to prepare thin specimens at the welded metal zone, as shown in Figure 7. Finally, we employed a field emission gun transmission electron microscope (Philips CM200), operating at 200 kV, equipped with energy-dispersive X-ray spectroscopy (EDS), on a copper grid specimen, followed by crystallographic data identification of the oxide inclusions using selected area diffraction pattern (SADP), as examined by bright-field imaging (BF-image).

**Figure 8:**
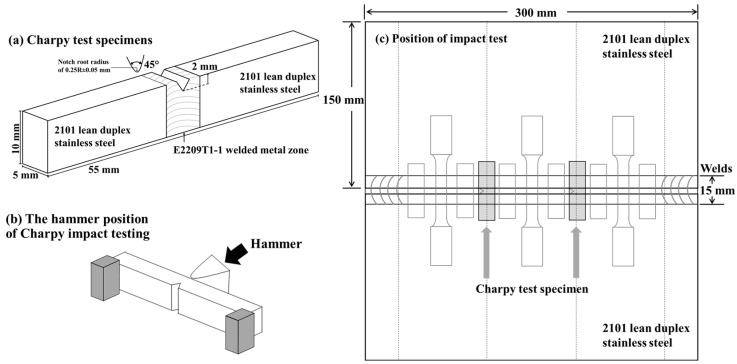
Schematic showing the Charpy v-notch impact testing process: (**a**) the test specimens; (**b**) the hammer position; (**c**) the specimen’s position for impact tests.

Next, we performed impact tests by Charpy impact tests at room temperature (28 ± 2 °C) by implementing the absorbed energy of a swinging hammer at 300 J and 5.24 m/s. First, the Charpy v-notch impact specimens were prepared with subsize thickness dimensions of 55 mm × 10 mm× 5 mm at a v-notch angle of 45°, notch root radius of 0.25R ± 0.05 mm, and a notch depth of 2 mm in the welded metal zone, following ASTM E23-18, as shown in Figure 8. Notably, we located the impact specimen at the center of the welded metal zone (width of the welded metal zone: 15 mm) to ensure the weld’s impact region, followed by testing the three specimens for impact toughness. Then, we characterized the fracture surfaces via secondary electron imaging in a high-resolution field emission gun SEM (JSM-IT800) to observe the fracture’s morphology.

## 4. Conclusions

The qualitative analyses of oxide inclusions in the newly developed flux-cored arc shows that welded 2101 lean duplex stainless steel is spherical, with the mixed oxides containing titanium-, chromium-, manganese-, and silicon-rich oxides. We also observed that although titanium dioxide formed earlier than manganese- and silicon-rich oxides in the form of a solid structure, manganese- and silicon-rich oxides formed as an amorphous structure because of their rapid cooling in the weld pool. Furthermore, the absorption energy from impact testing of the welded flux-cored arc 2101 using E2209T1-1 as the filler metal was comparable to other reports, even though oxide inclusions were found. However, these oxide inclusions (orthorhombic TiO_2_, tetragonal TiO_2_, cubic MnO, amorphous manganese, and amorphous silicon oxide) did not strongly affect crack initiation or propagation.

## Figures and Tables

**Table 1:** EDS point analysis of the oxides, corresponding to Figure 3 and Figure 4.

Element (wt.%)	Small Oxide		Large Oxide		
	SADP on Figure 3b–d	SADP on Figure 3e	SADP on Figure 4b,c	SADP on Figure 4d	SADP on Figure 4g
O	25.11	29.18	24.54	44.02	32.27
Si	0.46	24.97	0.45	47.17	24.83
Ti	15.62	0.11	20.94	0.17	1.88
Cr	29.25	3.28	23.36	2.89	3.55
Mn	27.28	35.14	27.57	0.43	30.30
Mo	0.11	3.59	0.00	0.13	1.90
Ni	0.19	0.27	0.26	0.54	0.22
Fe	1.72	2.30	2.45	4.65	2.05
Al	0.27	1.17	0.42	0.00	1.99

SADP: selected area diffraction pattern of TEM.

**Table 3:** Nominal chemical compositions of the stainless steel base material and filler metal.

Material and Chemical Composition (wt.%)	C	N	Mo	Si	Ni	Mn	Cr	Fe
2101 lean duplex stainless steel (UNS S32101)	<0.04	0.20–0.25	0.10–0.80	<1.00	1.35–1.70	4.00–6.00	21.00–22.00	Bal.
E2209T1-1 filler metal (AWS A5.22)	0.03	0.11	3.42	0.49	8.95	0.67	22.00	Bal.

## Data Availability

Not applicable.
